# Editorial: Is Early Onset of Alcohol Use Associated With Later Alcohol Use?

**DOI:** 10.3389/fnbeh.2020.00133

**Published:** 2020-08-13

**Authors:** Ricardo Marcos Pautassi, Adrian J. Bravo, Elio Acquas, Angelina Pilatti

**Affiliations:** ^1^Instituto de Investigación Médica M. y M. Ferreyra (INIMEC–CONICET-Universidad Nacional de Córdoba), Córdoba, Argentina; ^2^Facultad de Psicología, Universidad Nacional de Córdoba, Córdoba, Argentina; ^3^Department of Psychological Sciences William & Mary, Williamsburg, VA, United States; ^4^Department of Life and Environmental Sciences and Centre of Excellence on Neurobiology of Addiction, University of Cagliari, Cagliari, Italy; ^5^Instituto de Investigaciones Psicológicas, IIPsi-CONICET-UNC, Córdoba, Argentina

**Keywords:** alcohol, early onset, adolescents, adults, prenatal

Research in the neuroscience and epidemiology of alcohol use disorders (AUD) has significantly contributed to our understanding of why individuals use alcohol and why a significant fraction transitions to risky alcohol use or develop an AUD (Harris and Koob, 2017). Yet research efforts are still unraveling important scientific questions. One of those lingering, unanswered questions is the role that an early age of alcohol onset has on the probability of later engaging in risky drinking and/or developing an AUD. The promoting effect of an early alcohol onset on later alcohol use or AUD is an ubiquitous finding (Marshall, 2014). Whether or not the association implies a causal relationship is, however, still under investigation. It is possible that early alcohol exposure alters brain (Pascual et al., 2007) or social processes (Light et al., 2013) that, in turn, facilitate alcohol seeking and intake. Yet it is also possible that both events are explained by a common factor, such as a psychiatric precursor (Tedor et al., 2018). This Research Topic presents contributions toward bridging the gap between preclinical, clinical, and epidemiological research, highlighting new information to better understand the consequences of early alcohol exposure.

Binge or heavy episodic drinking (HED; ≥4 or 5 standard drinks in a short, usually ≤2 h, drinking occasion, for females and males, respectively) is a pattern of excessive alcohol intake prevalent in emerging adults and, particularly, in those with early drinking onset (Pilatti et al., 2017). The effects of binge drinking on the integrity of sub-cortical structures and on inhibitory control have been not extensively studied in humans. Moreover, there is dearth of longitudinal studies analyzing predictors of binge drinking in non-US or non-European samples. In this Research Topic Vera et al. identified, in 1240 Argentinian college students, six binge HED trajectories (Moderate Stable Frequency, Moderate Decreasing Frequency, Stable Infrequent, Decreasing Infrequent and No-HED), with membership in those trajectories with more frequent HED being promoted by a younger age of first drink or intoxication, greater perception of peer drinking frequency and higher levels of impulsivity. Two studies published on this Topic tested, via magnetic resonance imaging, binge-related alterations on the structural properties of the nucleus accumbens and caudate nucleus, and on the balance between response inhibition and alcohol-related processing. Suárez-Suárez et al. reported that, despite similar performance in an alcohol-cued Go/NoGo task, binge drinkers, but not their controls, showed increased frontal activity in the inferior frontal gyrus and in the anterior insula, probably reflecting a compensatory mechanism. The study by Sousa et al. revealed that the volume of the nucleus accumbens was increased in 20 college students (aged 18–23 years old) that reported having engaged in binge drinking at least once per month (with a minimum duration of 10 months), when compared to their abstinent peers. These studies make significant contributions to our understanding of predictors and consequences of binge drinking/HED in young adults.

Adolescent vs. adult differences in response to binge alcohol are dissected in the contributions of Rosana Camarini's and Ricardo Pautassi's groups. The study by Carrara-Nascimento et al. addresses the impact of a binge-like dose of alcohol, administered 5 days after a sub-chronic alcohol exposure regimen, on adolescent or adult mice. Despite similar alcohol-induced locomotor sensitization at both ages, the study revealed age-related differences in prefrontal cortical and accumbal dopamine content, such that levels were lower in adolescent mice than in adult mice. Salguero et al., in turn, assessed the impact of binge-like voluntary drinking during adolescence or young adulthood on anxiety response, shelter seeking, and recognition memory at late adolescence, as well as on voluntary alcohol drinking. The study disclosed that rats binge-drink more in adolescence than in adulthood and that this pattern negatively impacts anxiety responses in late adulthood. Moreover, adolescent exposure to alcohol enhanced alcohol consumption in adulthood.

Exciting data pinpoint alcohol-induced neuroinflammation as a relevant factor in the pathogenesis of AUD (Kelley and Dantzer, 2011). The mini-review by Flores-Bastías et al. puts forward the hypothesis that adolescent alcohol exposure reduces the availability of the α-melanocyte-stimulating hormone, and hence the activation of hippocampal melanocortin four receptors. This leads to reduced activity of the brain-derived neurotrophic factor, which is associated with reduced neurogenesis and neuroinflammation. The authors suggest that this signaling pathway may link adolescent alcohol exposure to greater likelihood of AUD in adulthood. The authors conclude that the activation of melanocortin four receptors (for instance, via synthetic agonist peptides) should rescue these effects. Mira et al., on this Topic, further review the detrimental effects of developmental alcohol exposure, including alcohol-induced impairments in neuronal morphology and survival, on hippocampal function. The authors review critical data, with a focus in glutamatergic transmission, suggesting that prenatal/perinatal or adolescent alcohol exposure is more harmful to hippocampus-dependent cognitive abilities than adult alcohol exposure.

The other mini-review of this Topic, that of Towner and Varlinskaya, reviews the pre-clinical literature on the effects of adolescent alcohol exposure on later alcohol acceptance and associated behavioral alterations. It establishes that many, yet not all, of the studies reviewed showed increases in voluntary alcohol intake after adolescent alcohol exposure. The authors pinpoint that those studies that yielded high levels of alcohol intoxication at adolescence were more likely to report enhanced alcohol drinking in adulthood, than those that induced moderate or low levels of alcohol intoxication in adolescence. This suggests that pre-clinical models of adolescent alcohol exposure should focus on preparations that induce relatively high blood alcohol levels.

The first exposure to binge alcohol can also occur during gestation (Yates et al., 1998; Baer et al., 2003). This prenatal alcohol exposure (PAE) may disrupt normative brain development by altering the functioning of the immune system (Gauthier, 2015). The study by Doremus-Fitzwater et al. on this Topic indicates that PAE (i.e., an alcohol liquid diet given on gestational days 6–20; 35% daily calories from alcohol) may potentiate postnatal expression of Il-6 and IkBa after an alcohol challenge. These intriguing results suggest that PAE may exert long-term alterations in the neuroimmune gene expression of neuroinflammatory factors. PAE has also been associated with increased alcohol intake in the offspring, which could be the result of PAE enhancing stress sensitivity or anxiety. The latter was supported by Madarnas et al. in one of the articles of this Topic. Moreover, Madarnas et al. found that the anxiety-prone phenotype induced by PAE (forced access to 6% alcohol, for 20 days prior to mating and throughout pregnancy and lactation) was associated with alterations in the radial distribution of axons of the cingulate cortex and in the expression of serotonergic and cannabinoid receptors.

The postnatal increase in alcohol intake found after PAE can also relate to early associative learning comprising alcohol's sensory properties and pharmacological effects (Spear and Molina, 2005). The review by Gaztañaga et al. makes a critical appraisal of this possibility. The authors make the case that the neural underpinnings of this prenatal appetitive learning rely on fetal brain acetaldehyde activating the endogenous opioid system. The paper contributed by Miranda-Morales et al. provides a comprehensive and insightful review of the literature on this and other critical topics, related to prenatal exposure to moderate concentrations of alcohol (e.g., intubations of 1.0 or 2.0 g/kg/day, on gestational days 17–20). The review discusses the role played during the intrauterine life by olfactory and gustatory cues (toward the development of detection and discrimination capabilities) and that of such exposure in the recognition of the reinforcing properties of alcohol through associative memories.

While much research has examined the impact that early drinking has on alcohol dependence, few has examined whether this link depends on cross-national drinking context and policies. In their study, Conde et al. found a moderate/strong positive association between early drinking and dependence among participants across 169 countries; however, the strength of this association was context-specific, based on normative drinking practices and linked to country level income and gender. Specifically, strong relationships between early drinking and alcohol dependence were present among countries where abstention or low infrequent drinking seems to be normative, while no significant relationships were found in countries where drinking is the common practice.

The findings and discussions presented in this Topic suggest that delaying age of first alcohol use is a deterrent of subsequent engagement in risky drinking and can protect from brain neurotoxicity and insult. Moreover, the first exposure to alcohol can occur much earlier than in adolescence, i.e., during prenatal life, which is associated with multiple neurobehavioral deficits and alterations in postnatal reactivity toward the drug.

## Author Contributions

All authors listed have made a substantial, direct and intellectual contribution to the work, and approved it for publication.

## Conflict of Interest

The authors declare that the research was conducted in the absence of any commercial or financial relationships that could be construed as a potential conflict of interest.
